# Thoracic aortic calcifications on chest radiographs and incident major adverse limb events in cardiovascular disease patients

**DOI:** 10.1007/s10554-025-03435-5

**Published:** 2025-06-02

**Authors:** Netanja I. Harlianto, Firdaus A.A. Mohamed Hoesein, Willem P.T.H. Mali, Marjolein E. Hol, Constantijn E.V.B. Hazenberg, Joost A. van Herwaarden, Wouter Foppen, Pim A. de Jong, UCC-SMART-Studygroup

**Affiliations:** 1 Department of Radiology, University Medical Center Utrecht and Utrecht University, Heidelberglaan 100, Utrecht, 3508 GA the Netherlands; 2 Department of Vascular Surgery, University Medical Center Utrecht and Utrecht University, Utrecht, the Netherlands

**Keywords:** Thoracic aortic calcification, Calcification, Atherosclerosis, Endpoints

## Abstract

Thoracic aortic calcifications (TAC) are a common finding in medical imaging. We assessed the association between TAC on chest radiographs and major adverse limb events (MALE), other cardiovascular outcomes, and mortality in cardiovascular disease patients. A total of 4680 patients were included from the prospective Utrecht Cardiovascular Cohort-Second Manifestation of ARTerial disease cohort. TAC severity was classified based on chest radiographs by certified readers as mild, moderate, and severe. Multivariate cox proportional hazard models were utilized to assess associations between TAC and incident MALE defined as major amputation, peripheral revascularization or thrombolysis in the lower limb. Secondary endpoints included major adverse cardiovascular events (MACE: stroke, myocardial infarction, and vascular death), and all-cause mortality. A total of 4680 patients were included. TAC was present in 1789 (38%) patients. After a median follow-up of 11.8 years (interquartile range: 7.6–15.7 years), 426 MALE, 992 MACE, and 1387 deaths occurred. TAC presence was associated with incident MALE after adjustments, (hazard ratio (HR):2.14;95%CI:1.73–2.65) and this risk increased with TAC severity (HR_mild_: 1.97; 95%CI:1.51–2.57, HR_moderate_: 2.04; 95%CI:1.56–2.68, and HR_severe_: 2.71; 95%CI: 2.02–3.65). Moreover, TAC was associated with incident MACE (HR:1.22;95%CI:1.06–1.40), ischemic stroke (HR: 1.45; 95%CI: 1.11–1.91), and vascular death (HR: 1.35; 95%CI: 1.13–1.63). TAC was only associated with all-cause mortality (HR:1.34;95%CI:1.18–1.52) in patients who experienced a previous cardiovascular event. In cardiovascular disease patients, TAC on chest radiographs is associated with an increased risk for incident MALE. In addition, we found that TAC was associated with incident ischemic stroke, MACE and all-cause mortality.

**Supplementary Information:**

The online version contains supplementary material available at 10.1007/s10554-025-03435-5.

## Introduction

Arterial calcification in the aorta results in hardening or stiffening and is histologically mainly fibrocalcific intimal disease and to a lesser extent medial disease [1, 2]. Thoracic aortic calcifications (TAC) are frequently observed as incidental findings on chest radiographs or computed tomography (CT) scans [3]. Chest radiographs are the most commonly ordered test in radiology, and the number of chest CTs is rising, providing insight into aortic calcification in a substantial proportion of the population [3].

TAC has been identified as an independent predictor for cardiovascular risk factors and for several outcomes [4, 8]. Prior studies on the prognostic implications of TAC have predominantly focused on (all-cause) mortality, ischemic stroke, and myocardial infarction [4, 8]. However, lower limb endpoints in relation to calcifications, especially those in the thoracic aorta, are understudied. Two prior studies have assessed lower limb endpoints in relation to TAC, both in populations without prior cardiovascular disease at the time of inclusion [9, 10]. Little is known about the association between TAC and major adverse limb events (MALE) in a high-risk cardiovascular disease cohort. A possible explanation of how TAC could be related to MALE is that TAC has shown to reflect distal aorta and the lower limb calcifications [11]. A recent cross-sectional study found that patients with severe TAC (> 400 mm) had a higher proportion of peripheral vascular disease [12]. Likewise, previous work has shown that patients with chronic limb-threatening ischemia (CLTI) have extensive calcification at other vascular beds [13], including in the thoracic aorta.

The aim of this prospective study was to assess the association between TAC as measured on chest radiographs and the occurrence of MALE in patients with cardiovascular disease. Secondary aims were to assess the relation with TAC and major adverse cardiovascular events (MACE), individual MACE components, and all-cause mortality.

## Materials and methods

### Study population

The Utrecht Cardiovascular Cohort - Second Manifestations of ARTerial disease (UCC-SMART) study, is an ongoing prospective cohort study which started in 1996, following patients between the ages of 18 and 79 years with either manifest or risk factors for vascular disease. The UCC-SMART study adhered to the Declaration of Helsinki, was approved by the medical ethics committee of the UMC Utrecht, and all included patients gave written informed consent [14]. For the current study we included patients with a digital chest radiograph within three months of inclusion in the UCC-SMART study.

### Physical and laboratory measurements

All included patients in the UCC-SMART study underwent extensive vascular screening [14]. Screening included a health questionnaire covering medical history, risk factors, smoking and drinking habits, and prescribed drugs. In addition, physical examination and laboratory testing in a fasting state were performed according to a standard diagnostic protocol [14].

Blood pressure was measured using a non-random sphygmomanometer which was performed three times simultaneously at the right and left upper arm in an upright position with an interval of 30s. The mean of the last two measurements from the highest arm was used. Hypertension was defined as systolic blood pressure ≥ 140 mmHg and/or diastolic blood pressure ≥ 90 mmHg and/or use of antihypertensive medication. Body mass index (BMI) was calculated as weight divided by height squared (kg/m2). Fasting blood samples were available for blood lipids, HbA1c, glucose, high-sensitive C-reactive protein (hsCRP), and creatinine levels [14]. Renal function was estimated using the Chronic Kidney Disease Epidemiology Collaboration equation [15]. Diabetes mellitus at baseline was defined as either a referral diagnosis of diabetes, self-reported diabetes including use of glucose-lowering agents, glucose ≥ 11.1 mmol/L, or initiation of glucose lowering treatment within one year after inclusion with a glucose ≥ 7.0 mmol/L at baseline [14]. Hyperlipidemia was defined as low-density lipoprotein (LDL)-cholesterol ≥ 2.6 mmol/L [16]. The metabolic syndrome was defined according to the National Cholesterol Education Program criteria [17].

### TAC assessment

**Fig. 1:**
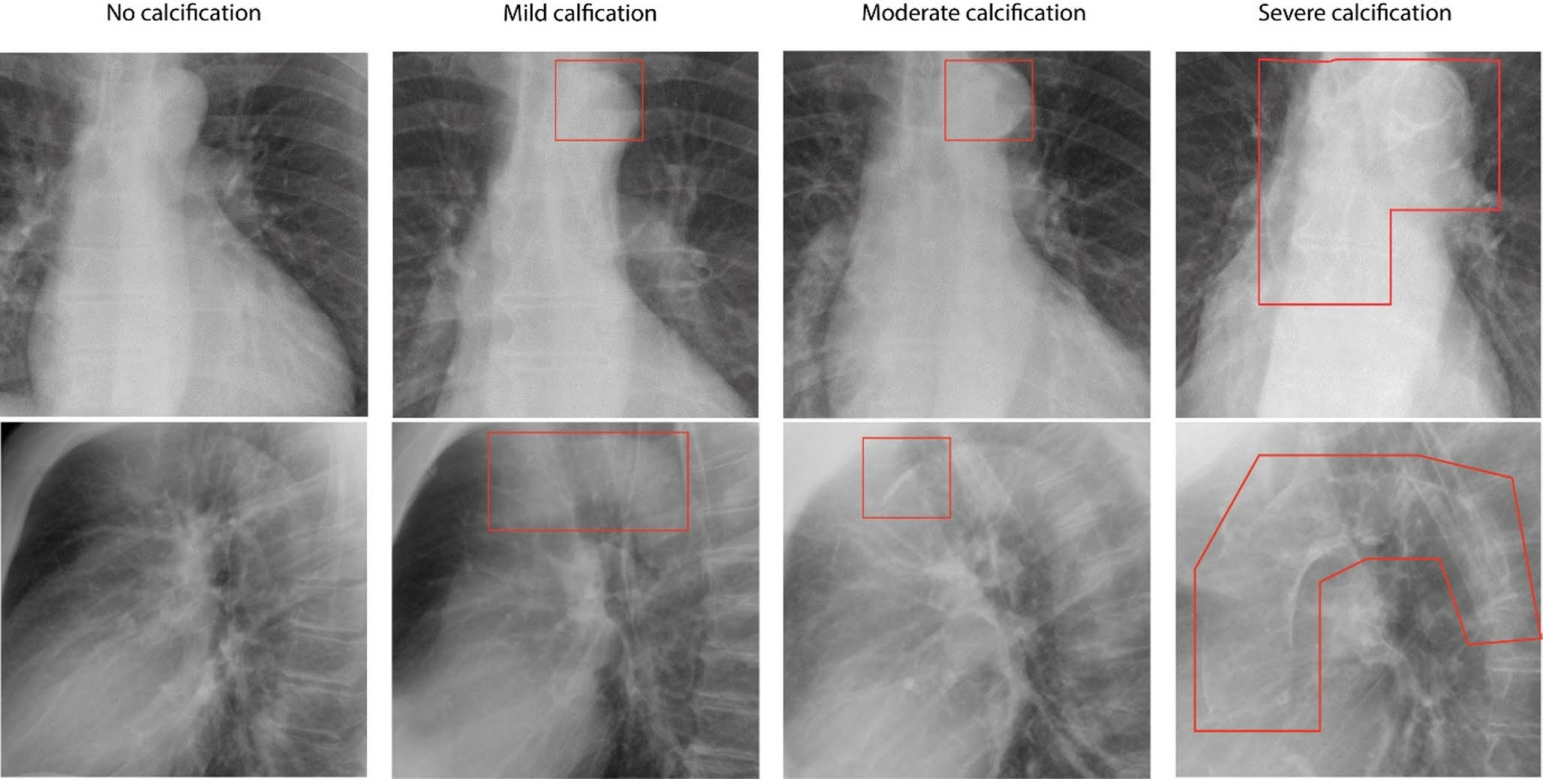
TAC severity example

TAC assessment has been previously described and illustrated [18]. Briefly, TAC presence and severity was visually graded on chest radiographs by one of six certified readers (two radiologists and four radiology residents with Entrustable Professional Activity level 4 or 5 for chest radiograph interpretation) from the department of Radiology of our institution. TAC severity was classified as the following: no TAC: no visible calcifications; mild TAC: borderline calcifications or mild calcification suspected; moderate TAC: clear calcification, multiple dots or one large calcification; severe TAC: extensive calcification. Readers were blinded to patient characteristics and outcomes (Fig. 1).

### Endpoint assessment and follow-up

Patients were requested to complete biannual questionnaires regarding outpatient clinical visits and hospitalizations. In the case of a possible event, data from the general practitioner, results of relevant laboratory and imaging examinations, and hospital discharge letters or other correspondence were retrieved. Hereafter, all events were audited by three independent physicians of the SMART endpoint committee from different departments. Any disagreement was discussed with other Endpoint Committee members, and the majority of the classifications received as the basis for the ultimate decision. The period between enrolment to death from any cause, the occurrence of an event, date of loss to follow-up, or the preselected date of January 2023 was defined as follow-up. The primary endpoint of our study included major adverse limb events (MALE) defined as lower limb revascularization (vascular intervention or thrombolysis) and major amputation (at the level of the ankle or more proximal). Secondary endpoints were major adverse cardiovascular events (MACE: non-fatal myocardial infarction, non-fatal stroke and vascular death), and all-cause mortality.

### Data analysis

Data analysis was performed with R, version 4.3.3 (R Foundation for Statistical Computing, Vienna, Austria). Statistical significance was set at *p* < 0.05. Normal distributed data were expressed using the mean and standard deviation, and categorical variables using frequencies and percentages. Differences between groups with and without TAC were assessed with age and sex adjusted logistic regression, stated as odds ratios (OR) with 95% confidence intervals (95%CI). Multivariate cox proportional hazard models were used to assess the associations between TAC and cardiovascular endpoints and all-cause mortality expressed as hazard ratio (HR) with 95%CI. Adjustments were made for age sex, BMI, renal function, systolic blood pressure, diabetes, smoking status, and non-high-density lipoprotein (HDL) cholesterol. Using Schoenfeld residuals, the proportional hazard assumption was evaluated, which was not violated. Adjusted Kaplan-Meier curves were assessed using the direct standardization method between TAC and MALE. In sensitivity analysis, we performed analyses adjusted for competing risks of mortality, using Fine and Gray regression modeling. Moreover, we performed subgroup analyses after excluding subjects with a history of peripheral artery disease (PAD) or a history of stroke, and diabetes mellitus. We also adjusted cox regression models additionally for hsCRP and anticoagulation usage. Finally, we performed additional analyses to evaluate the interaction effects between TAC and age, sex, diabetes and smoking status. Missing covariate data for BMI (0.1%), systolic blood pressure (0.1%), packyears (0.1%), non-HDL cholesterol (0.3%), and renal function (0.3%) were imputed by means of single regression imputation with the “mice” package. Data are reported according to the STROBE (STrengthening the Reporting of OBservational studies in Epidemiology) Guidelines [19].

## Results

### Patient characteristics

A total of 4791 patients were identified, of whom 167 were excluded for technical image deficiencies (*n* = 44), only AP radiograph available (*n* = 34), poor image quality (*n* = 10) and due to missing follow-up (*n* = 23). Finally, a total of 4680 patients were included, with a mean age of 58.4 years (standard deviation: 11.2) of which 69.7% was male. 38% of patients had evidence of TAC, which consisted of 725 patients with mild TAC, 652 patients with moderate TAC, and 409 patients with severe TAC. Baseline patient characteristics between patients with and without TAC are provided in Table 1. Patients with TAC presence were older (63.8 vs. 55.0 years), more often female (35.3% vs. 27.3%), had a higher proportion of diabetes mellitus (24.7% vs. 19.9%), had a higher history of smoking (77.7% vs. 70.5%), and history of vascular disease (80.1% vs. 66.1%).

**Table 1:** Baseline patient characteristics

	No TAC (*n* = 2894)	TAC (*n* = 1786)	Adjusted odds ratio (95%CI)	Adjusted *p*-value
Age, mean (SD),- years	55.0 (11.1)	63.8 (9.0)	1.09 (1.09–1.10)	< 0.001
Female sex, no (%)	790 (27.3)	630 (35.3)	1.79 (1.55–2.07)	< 0.001
BMI, mean (SD), kg/m^2^	27.2 (4.6)	26.9 (4.3)	0.97 (0.95–0.98)	0.007
Systolic blood pressure, mean (SD), mmHg	139.4 (21)	144.4 (21.8)	1.00 (1.00-1.01)	0.004
Hypertension, no (%)	692 (23.9)	473 (26.5)	1.08 (0.92–1.25)	0.38
Diabetes mellitus, no ( %)	576 (19.9)	441 (24.7)	1.16 (0.99–1.35)	0.07
Glucose, mean (SD), mmol/L	6.3 (1.8)	6.5 (1.9)	1.03 (1.01–1.08)	0.02
HbA1c, mean (SD), %	5.9 (1.0)	6.1 (1.1)	1.09 (1.01–1.17)	0.01
Non-HDL, mean (SD), mmol/L	3.6 (1.3)	3.7 (1.3)	1.11 (1.06–1.17)	< 0.001
Hyperlipidemia, no (%)	1247 (43.1)	772 (42.3)	1.21 (1.06–1.39)	0.005
CKDEPI, mean (SD)	81.1 (19.1)	73.9 (18.4)	1.00 (0.99–1.01)	0.09
High sensitive C-reactive protein, mean (SD), mg/L	4.1 (7.8)	4.6 (8.9)	1.00 (0.99–1.01)	0.31
Metabolic syndrome, no (%)	1534 (53.0)	995 (55.7)	1.10 (0.94–1.28)	0.18
Ever smoking, no (%)	2040 (70.5)	1388 (77.7)	1.61 (1.38–1.88)	< 0.001
Packyears, mean (SD), years	15.7 (18.2)	20.5 (21.0)	1.01 (1.01–1.02)	< 0.001
Antiplatelet therapy	1668 (57.6)	1184 (66.2)	1.01 (0.88–1.16)	0.88
Oral anticoagulants	230 (7.9)	216 (12.1)	1.19 (0.96–1.47)	0.11
History of any cardiovascular disease, no (%)	1913 (66.1)	1431 (80.1)	1.58 (1.33–1.89)	< 0.001
History of PAD, no (%)	197 (6.8)	279 (15.6)	2.47 (2.00-3.05)	< 0.001
History of CAD, no (%)	1346 (46.5)	927 (51.9)	0.91 (0.79–1.04)	0.17
History of cerebrovascular disease, no (%)	391 (13.5)	336 (18.8)	1.26 (1.06–1.50)	0.008

Data are shown as mean (standard deviation) or frequency (percentage). Logistic regression for any TAC adjusted for age, sex. TAC: thoracic aortic calcification; SD: standard deviation. 95%CI: 95% confidence interval. PAD: peripheral artery disease; CAD: coronary artery disease; CKDEPI: Chronic Kidney Disease Epidemiology Collaboration

### Outcomes and follow-up

After a median follow-up of 11.8 years (7.6–15.7 years), 1387 patients had died of which 525 deaths were due to a cardiovascular cause. During the follow-up period, a total of 426 MALE and 992 MACE events occurred.

**Fig. 2:**
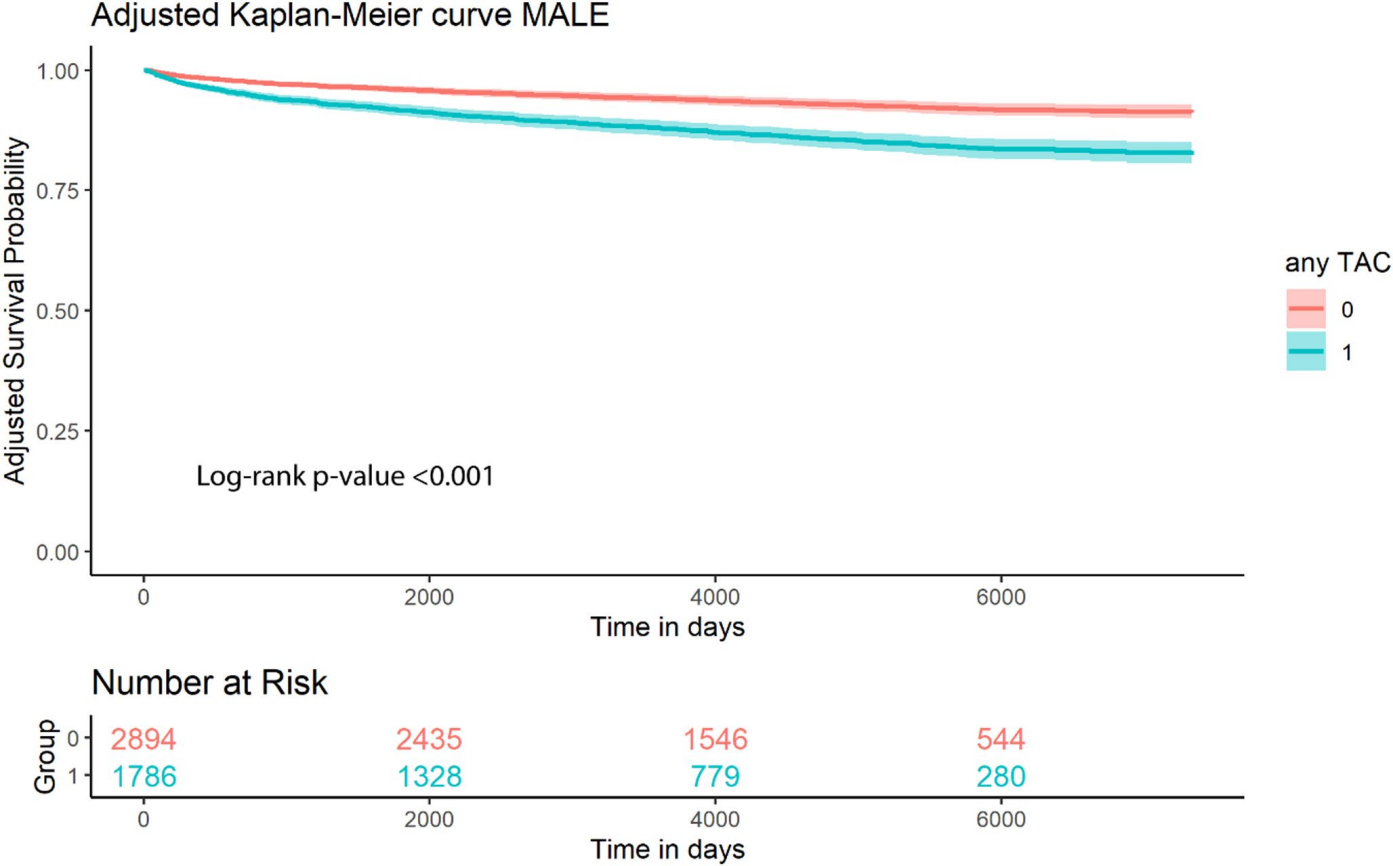
Adjusted Kaplan-Meier plot for major adverse limb events stratified by any thoracic aortic calcification. Model adjusted for age, sex, BMI, diabetes, systolic blood pressure, non-HDL cholesterol, kidney function, and packyears

The presence of any TAC was significantly associated with the occurrence of MALE (adjusted HR: 2.14; 95%CI: 1.73–2.65). Mild TAC (adjusted HR: 1.97; 95%CI:1.51–2.57), moderate TAC (adjusted HR: 2.04; 95%CI:1.56–2.68), and severe TAC (adjusted HR: 2.71; 95%CI: 2.02–3.65) were associated with the occurrence of MALE. The adjusted Kaplan-Meier curves using the cox model with direct adjustment in provided in Fig. 2.

Moreover, any TAC was significantly but more weakly associated with MACE (HR: 1.22; 95%CI: 1.06–1.40), ischemic stroke (HR:1.45; 95%CI: 1.11–1.91), vascular death (HR:1.35; 95%CI: 1.13–1.63), and all-cause mortality (HR 1.27; 95%CI: 1.14–1.43), after adjustment for confounders. There was no significant association between the presence of TAC and the occurrence of myocardial infarction. Cox regression analyses are provided in Table 2. Cumulative incidence functions between the presence and absence of TAC are provided in Supplementary Fig. 1–6.

**Table 2:** Cox regression modelling of thoracic aortic calcification and cardiovascular endpoints

	No. events	FU person years	Any TAC	TAC mild	TAC moderate	TAC severe
			HR (95%CI)	HR (95%CI)	HR (95%CI)	HR (95%CI)
MALE	426	51,360	2.14 (1.73–2.65)	1.97 (1.51–2.57)	2.04 (1.56–2.68)	2.71 (2.02–3.65)
All-cause mortality	1387	55,177	1.27 (1.14–1.43)	1.29 (1.11–1.49)	1.20 (1.04–1.39)	1.38 (1.17–1.62)
MACE	992	51,013	1.22 (1.06–1.40)	1.14 (0.95–1.36)	1.12 (0.93–1.34)	1.57 (1.29–1.91)
Vascular death	525	55,177	1.35 (1.13–1.63)	1.43 (1.13–1.81)	1.12 (0.87–1.43)	1.62 (1.26–2.09)
Ischemic stroke	245	53,346	1.45 (1.11–1.91)	1.28 (0.90–1.84)	1.58 (1.12–2.23)	1.56 (1.04–2.36)
Myocardial infarction	364	52,279	0.95 (0.76–1.19)	0.84 (0.62–1.16)	0.98 (0.72–1.33)	1.13 (0.79–1.63)

All cox models adjusted for age, sex, BMI, diabetes, systolic blood pressure, non-HDL cholesterol, kidney function, and packyears. P-value < 0.05 indicated in bold. FU: follow-up; TAC: thoracic aortic calcification. MALE: major adverse limb events; MACE: major adverse cardiac events

### Sensitivity analyses

Results between the presence of TAC and significant endpoints remained unchanged in sensitivity analyses adjusting for competing mortality risk (Supplementary Table 1), as well when additionally adjusting for hsCRP and anticoagulation usage. Likewise, after excluding 728 subjects with a history of PAD, the presence of TAC remained significantly associated with the occurrence incident of MALE for any TAC (HR: 2.45; 95%CI: 1.81–3.31), mild TAC (HR: 2.25; 95%CI: 1.55–3.27), moderate TAC (HR: 2.57; 95%CI: 1.78–3.71), and severe TAC (HR: 2.69; 95%CI: 1.72–4.19). Following the exclusion of 475 subjects with a history of cerebrovascular events, the presence of any TAC was also associated with the occurrence of incident ischemic stroke (HR 1.50; 95%CI: 1.09–2.09). After excluding patients with diabetes, TAC remained associated with incident MALE in multivariate analyses (HR: 2.28; 95%CI: 1.77–2.94). The relation between MALE and TAC was not affected after including separate interaction terms for age, sex, smoking status, diabetes and a history of vascular disease in the final adjusted model. The relation between TAC and all-cause mortality was influenced by a history of vascular disease (P- interaction = 0.005) (Supplementary Table 2). Subgroup analyses showed that the presence and severity of TAC was not associated with all-cause mortality in subjects without a history of vascular disease (HR: 0.87; 95%CI: 0.66–1.16). In contrast, in subjects with a history of vascular disease, TAC presence (HR: 1.34; 95%CI: 1.18–1.52) and severity (HR_mild_ TAC: 1.32; 95%CI: 1.12–1.56; HR_moderate_ TAC: 1.28; 95%CI: 1.09–1.51; HR_severe_ TAC: 1.46; 95%CI: 1.22–1.75) was significantly associated with all-cause mortality. (Supplementary Table 3).

## Discussion

This study aimed to evaluate TAC presence and severity on chest radiographs and its association with MALE, other incident cardiovascular events, and mortality in a population of cardiovascular disease patients. We found that the presence of TAC was associated with a more than doubled risk for MALE, and the risk increased with higher TAC severity, after extensive correction for cardiovascular risk factors. This relation remained robust in subgroup and sensitivity analyses. Whether patients with TAC require screening for peripheral arterial disease or alterations in treatment requires further investigation.

We found that TAC was associated with incident ischemic stroke, also for subjects who did not have a previous cerebrovascular event, corresponding favorably to previous literature. TAC was also associated with all-cause mortality in subjects who had experienced a previous cardiovascular event, which is also in line with prior studies [3], including the MESA [20], Rotterdam Study [21], and Framingham Heart Study [22]. While these endpoints have been extensively studied, the literature on MALE is limited, which has gained more interest in part due to growing prevalence of diabetes mellitus and poor outcomes associated with MALE [23, 24]. Guidelines propose therapy for peripheral arterial disease, which can aid prevention of hospitalization and amputation, if initiated timely [23]. However, a better understanding of the disease etiology and early detection remain important. Few previous studies have evaluated TAC in relation to lower limb outcomes. In the Multi-Ethnic Study of Atherosclerosis (MESA) it was observed that TAC measured on CT predicted incident clinical PAD, which was independent of traditional cardiovascular risk factors [9]. A single unit increase log transformed TAC was independently associated with of 13% higher risk for incident PAD. In contrast, another study using population data from 1964 to 1997 found no association between aortic arch calcifications on chest radiographs and peripheral vascular disease after multivariate adjustments [10]. We investigated a high risk population, in which we observed a clear and strong association, which adds to the literature and provides support for the observation in the MESA study.

Although our study does not provide conclusive evidence on a causal pathway from TAC to cardiovascular events, we will discuss several potential mechanisms. The strong association of TAC with cardiovascular events, including MALE, may be explained by the fact that TAC is a proxy of systemic arterial calcification, of arterial stiffening [24] or related to embolic disease [25]. First, there is substantial evidence that aortic calcification is closely linked to systemic atherosclerosis and arterial calcifications in other beds. For example, Wong and colleagues showed in MESA that aortic calcification was closely linked to atherosclerosis in the carotid, coronary, and lower limb arteries [26]. Others showed that aortic calcification was linked to coronary artery and carotid artery calcification [27, 28]. Despite the fact of substantial evidence on systemic atherosclerosis and arterial calcification, there is currently insufficient clinical evidence on whether the calcifications are a relevant treatment target, beyond the standard atherosclerosis treatment. Second, there is ample evidence that aortic calcifications are associated with an increased risk for distal thromboembolic events, especially cerebral [3, 29]. Whether the presence of TAC necessitates more aggressive (antithrombotic) therapy in the context of MALE or stroke is not clear and requires further study [30, 31]. Third, aortic calcifications are closely linked to increased pulse wave velocity and arterial stiffening [32–34]. Normal arterial hemodynamics are impacted by extensive aorta calcification through a dysfunctional Windkessel effect. leading to chronic damage in the brain, kidneys and the lower limbs [35]. For stroke, the link between stiffness of the siphon and distal vertebral arteries has indeed been found to be the strongest predictor of cerebrovascular events [36, 37]. Arterial stiffness is also important in relation to MALE. For example, CTLI patients have a higher arterial stiffness and incompressible crural arteries [38]. This arterial stiffness can predict amputation and death in CTLI patients [39].

Although arterial stiffness is difficult to assess and treat in clinical practice, it remains an important for our understanding of arterial (patho)physiology. The exact clinical consequences of the relation between TAC, arterial stiffness and MALE requires further study.

The strengths of the current study are its prospective design with uniform data collection in a large cardiovascular cohort. Second, we had extensive data on cardiovascular determinants which allowed for extensive adjustment in analyses. Finally, our population had long and complete follow-up with endpoint adjudication, which reduced subjectivity of endpoint assessment. Limitations include that the selection of patients with a clinically indicated chest radiograph may have increased the selection of higher risk subjects from the UCC-SMART cohort. Caution is therefore needed with generalizing our findings to lower risk populations. Finally, we had no data available on the interrater reliability of readers. It could be that noise from observer variation lowered our effect estimates However, the readers were certified to independently assess radiographs and our readings reflect clinical practice when such visual scoring systems would be implemented. Ideally, scoring of TAC would be done with automated methods in the future.

## Conclusion

The presence of TAC on chest radiographs was strongly associated with multiple cardiovascular endpoints, including MALE, in our cardiovascular disease patients, suggesting that calcifications of thoracic aorta may be a reflection of systemic atherosclerosis, thrombo-emboli and/or arterial stiffening. Whether incidental TAC should initiate screening of other vascular beds or should lead to treatment alterations requires further study.

## Electronic supplementary material

Below is the link to the electronic supplementary material.

Supplementary Material 1

## Data Availability

The informed consent that was signed by the study participants is not compliant with publishing individual data in an open access institutional repository or as supporting information files with the published paper. However, a data request can be sent to the SMART Steering Committee at uccdatarequest@umcutrecht.nl.
