# Supplementary material for: Thoracic aortic calcifications on chest radiographs and incident major adverse limb events in cardiovascular disease patients
Source: Int J Cardiovasc Imaging. 2025 Jun 2;41(7):1437–44. doi: 10.1007/s10554-025-03435-5 (PMC12241139; doi:10.1007/s10554-025-03435-5)
Supplement: Supplementary file 1 — Supplementary Material 1 [file 10554_2025_3435_MOESM1_ESM.docx]

**Supplementary Materials:**

Supplemental Table 1. Fine and Gray regression thoracic aortic calcification and cardiovascular endpoints

|  | **Any TAC** | **TAC mild** | **TAC moderate** | **TAC severe** |
| --- | --- | --- | --- | --- |
|  | **Subdistribution HR (95%CI)** | **Subdistribution HR (95%CI)** | **Subdistribution HR (95%CI)** | **Subdistribution HR (95%CI)** |
| MALE | **2.17 (1.74-2.70)** | **1.95 (1.48-2.56)** | **2.10 (1.59-2.78)** | **2.78 (2.05-3.78)** |
| MACE | **1.24 (1.09-1.42)** | 1.14 (0.96-1.36) | 1.15 (0.96-1.38) | **1.58 (1.30-1.93)** |
| Ischemic stroke | **1.45 (1.11-1.91)** | 1.27 (0.89-1.81) | **1.61 (1.14-2.29)** | **1.54 (1.01-2.36)** |
| Myocardial infarction | 0.94 (0.75-1.18) | 0.83 (0.61-1.13) | 0.99 (0.72-1.35) | 1.10 (0.76-1.58) |

Competing risks for mortality. All Fine and Gray models adjusted for age, sex, BMI, diabetes, systolic blood pressure, non-HDL cholesterol, kidney function, and packyears. P-value<0.05 indicated in bold.
FU: follow-up; TAC: thoracic aortic calcification. MALE: major adverse limb events; MACE: major adverse cardiac events.

Supplemental Table 2. Sensitivity analyses for age, sex, smoking, diabetes and history of vascular disease

|  | **Interaction term** | | | |  |
| --- | --- | --- | --- | --- | --- |
|  | **Age** | **Sex** | **Smoking** | **Diabetes** | **Vascular disease** |
|  | p-value | p-value | p-value |  | p-value |
| All-cause mortality | 0.61 | 0.87 | 0.38 | 0.12 | **0.005** |
| MACE | 0.07 | 0.46 | 0.46 | 0.95 | 0.36 |
| Vascular death | 0.85 | 0.70 | 0.96 | 0.22 | 0.28 |
| Ischemic stroke | 0.45 | 0.13 | 0.09 | 0.11 | 0.06 |
| MALE | 0.11 | 0.46 | 0.59 | 0.38 | 0.52 |

Models adjusted for age, sex, BMI, diabetes, systolic blood pressure, non-HDL cholesterol, kidney function, and packyears. MALE: major adverse limb events; MACE: major adverse cardiac events.

Supplemental Table 3. Cox regression all-cause mortality stratified by history of vascular disease.

|  | **Any TAC** | **TAC mild** | **TAC moderate** | **TAC severe** |
| --- | --- | --- | --- | --- |
|  | **HR (95%CI)** | **HR (95%CI)** | **HR (95%CI)** | **HR (95%CI)** |
| All-cause mortality (history of vascular disease = no) | 0.87 (0.66-1.16) | 1.04 (0.72-1.50) | 0.66 (0.43-1.01) | 0.94 (0.63-1.42) |
| All-cause mortality (history of vascular disease = yes) | 1.34 (1.18-1.52) | 1.32 (1.12-1.56) | 1.28 (1.09-1.51) | 1.46 (1.22-1.75) |

Cox regression for all-cause mortality in subjects without a history of vascular disease (N=1278) and with a history of vascular disease (N=3198). Models adjusted for age, sex, BMI, diabetes, systolic blood pressure, non-HDL cholesterol, kidney function, and packyears. TAC = thoracic aortic calcification.


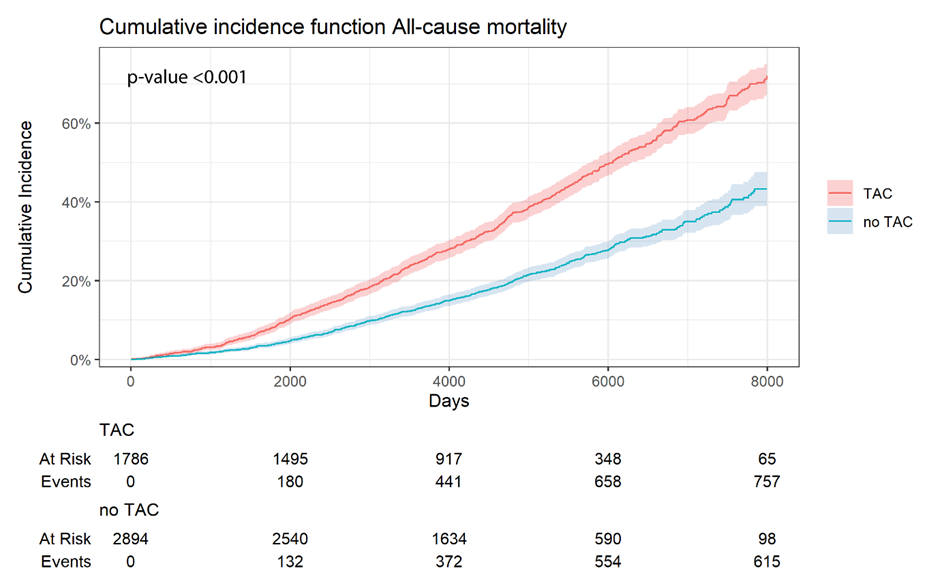


Figure 1. Cumulative incidence function all-cause mortality


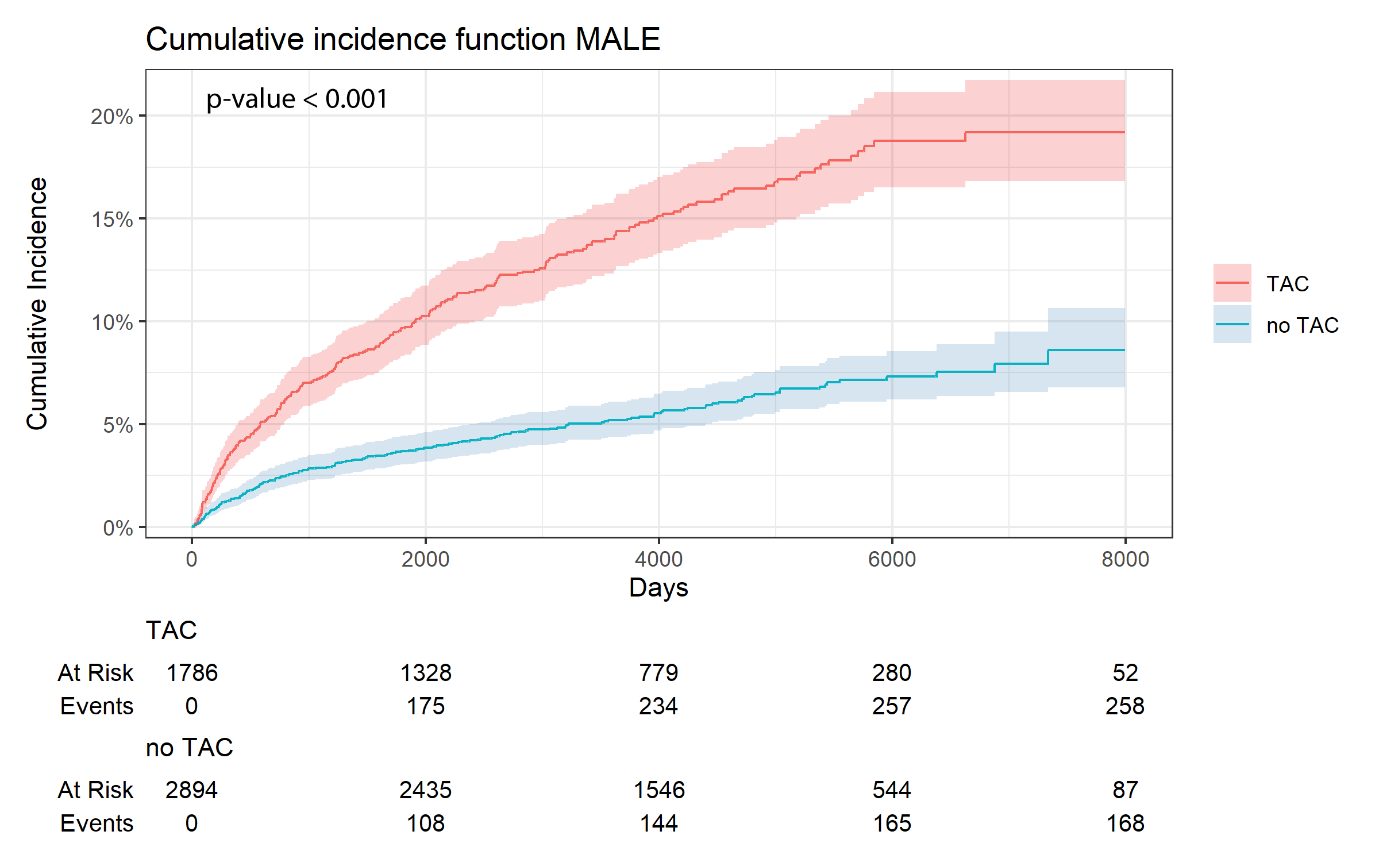


Figure 2. Cumulative incidence function major adverse limb events


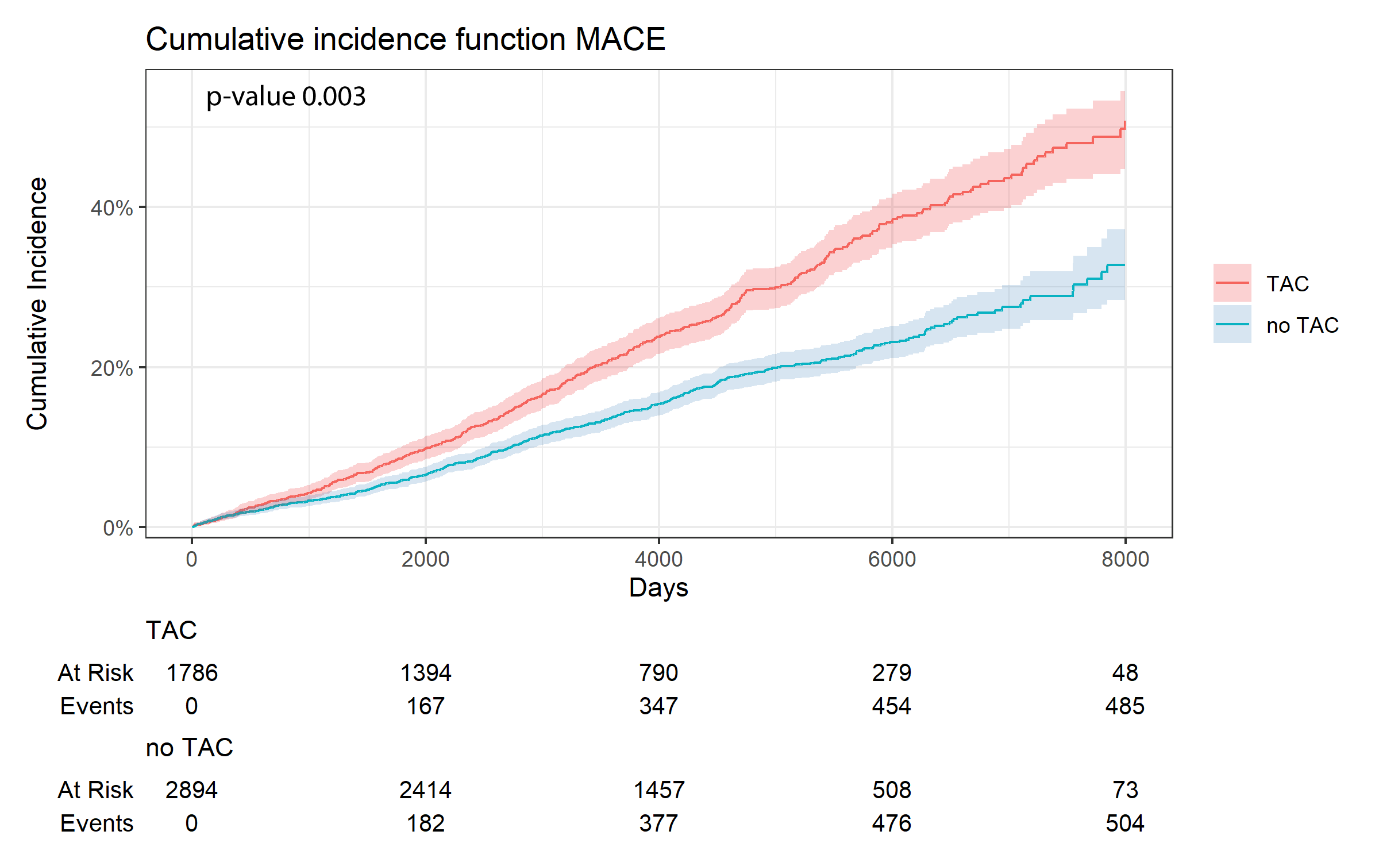


Figure 3. Cumulative incidence function major adverse cardiac events


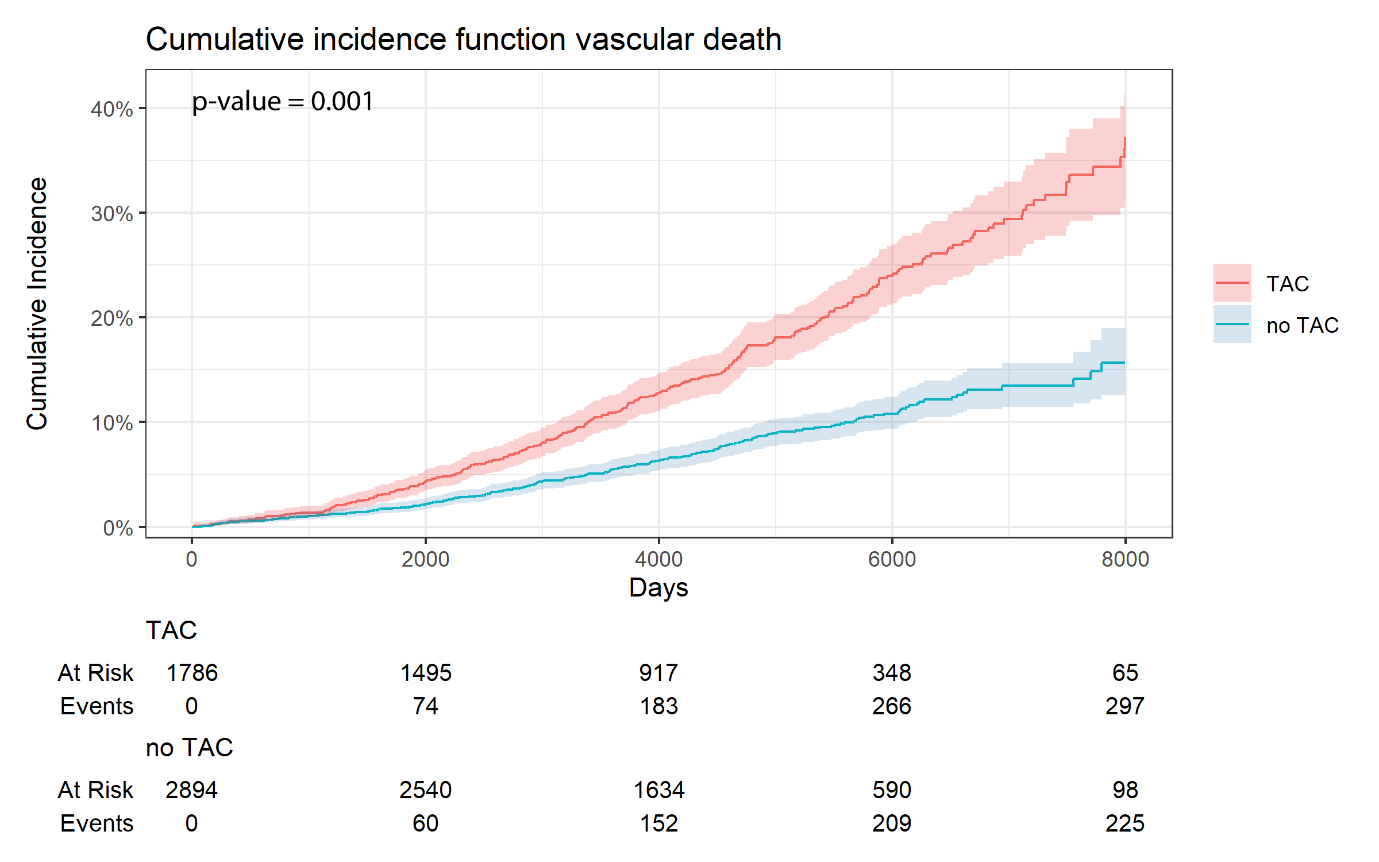


Figure 4. Cumulative incidence function vascular death


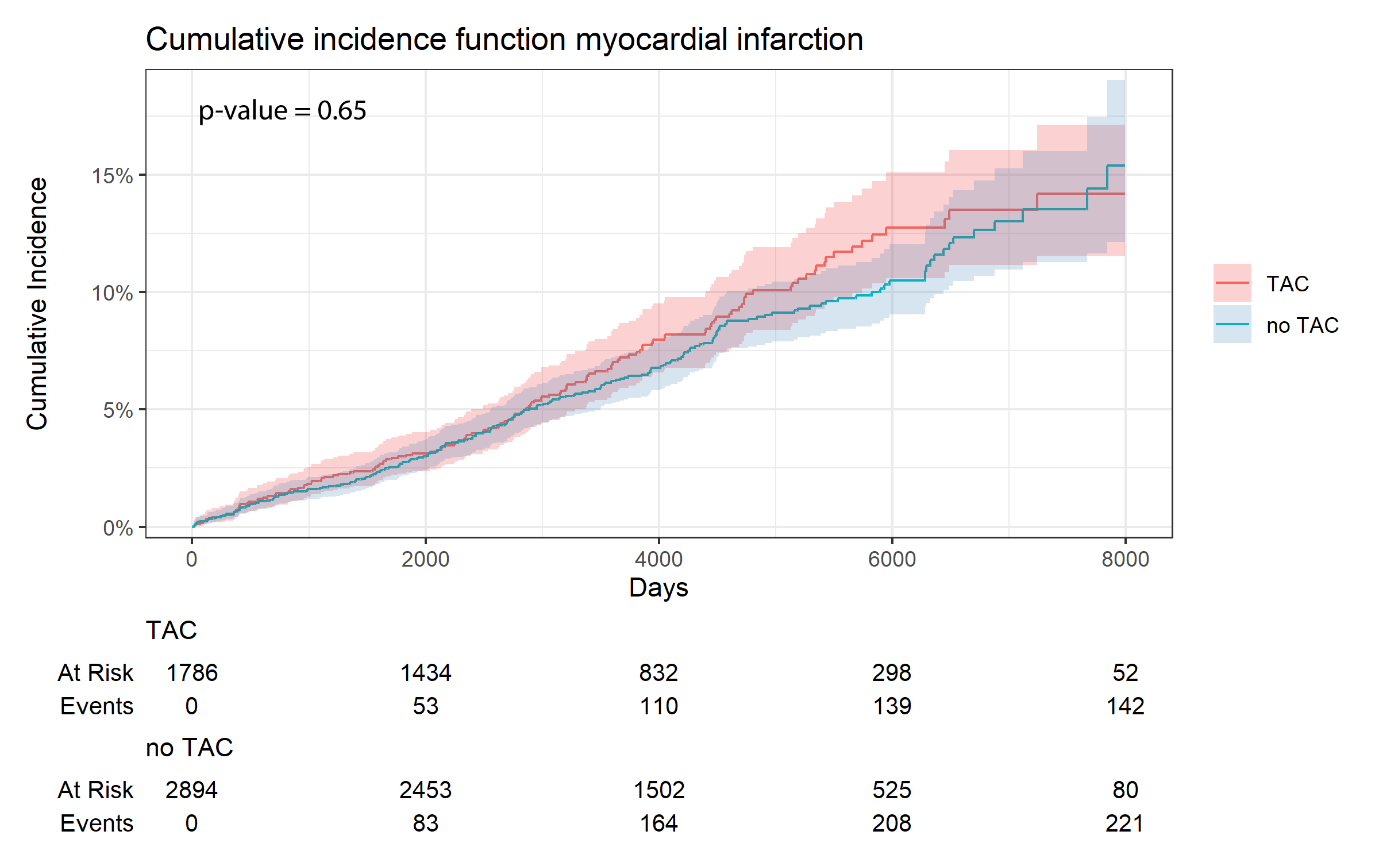


Figure 5. Cumulative incidence function ischemic stroke


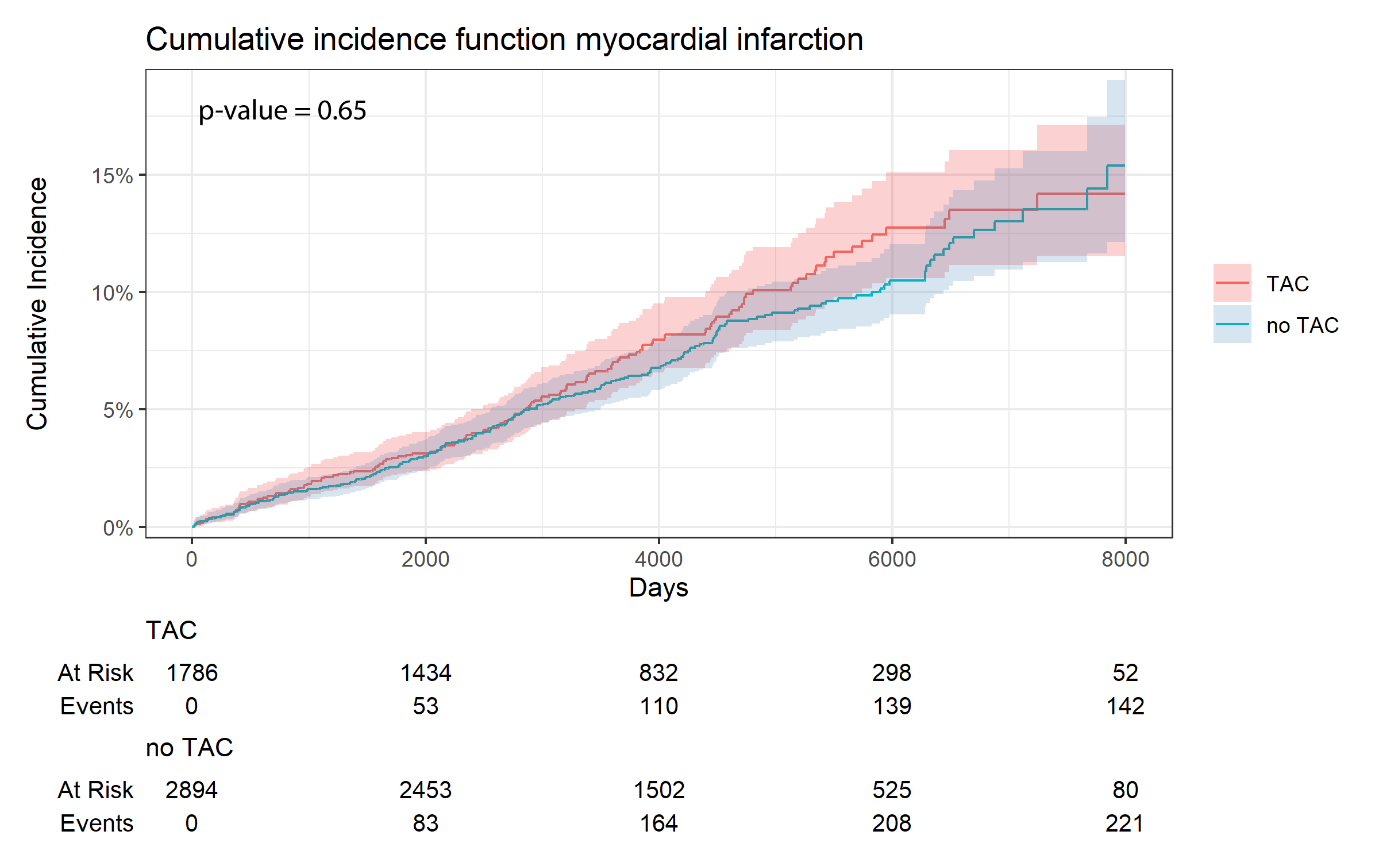


Figure 6. Cumulative incidence function myocardial infarction

First diagnosis at the time of inclusion:

Initial diagnosis/operation at the time of inclusion: cerebrovascular disease (N=471), peripheral vascular disease (N=350), coronary bypass grafting (N=459), percutaneous coronary intervention (N=1350), hypertension (N=741), hyperlipidemia (N=330), diabetes mellitus (N=285), Abdominal aorta aneurysm (N=106), ischemic renal disease (N=52), hypertensive pregnancy disorders (N=21), chronic HIV infection as a cardiovascular risk increasing condition (N=225), unspecified (N=276).
